# Efficacy and Safety of Radiosurgery in Cavernous Sinus Meningioma: A Systematic Review and Single Meta-Analysis

**DOI:** 10.1227/neuprac.0000000000000187

**Published:** 2025-11-06

**Authors:** Lucca B. Palavani, Raphael Camerotte, Marina Vilardo, Bernardo Vieira Nogueira, Lucas Pari Mitre, Paulo Victor Zattar Ribeiro, Henrique Lepine, Leonardo B. O. Brenner, Filipi Fim Andreão, Fábio Torregrossa, Thiago Scharth Montenegro, Raphael Bertani, Fábio Ynoe Moraes, Marcos Vinicius Calfat Maldaun, Cleiton Formentin

**Affiliations:** 1 Department of Neurosurgery, Max Planck University Center, Indaiatuba, São Paulo, Brazil;; 2 Department of Neurosurgery, Federal University of Rio de Janeiro, Rio de Janeiro, Brazil;; 3 Department of Neurosurgery, Catholic University of Brasília, Brasília, Brazil;; 4 Department of Neurosurgery, Serra dos Órgãos University Center, Teresópolis, Rio de Janeiro, Brazil;; 5 Faculty of Medicine, Santa Casa de São Paulo School of Medical Sciences, São Paulo, Brazil;; 6 Department of Medical Genetics, State University of São Paulo, Ribeirão Preto, São Paulo, Brazil;; 7 Department of Neurosurgery, University of São Paulo School of Medicine (FMUSP), São Paulo, Brazil;; 8 Department of Neurosurgery, State University of Ponta Grossa, Ponta Grossa, Paraná, Brazil;; 9 Department of Neurologic Surgery, Mayo Clinic, Rochester, Minnesota, USA;; 10 Departments of Neurosurgery and Otolaryngology—Head and Neck Surgery, Mayo Clinic Rhoton Neurosurgery and Otolaryngology Surgical Anatomy Program, Rochester, Minnesota, USA;; 11 Department of Neurosurgery, Spectrum Health/Michigan State University, Grand Rapids, Michigan, USA;; 12 Department of Neurosurgery, University of São Paulo, São Paulo, Brazil;; 13 Department of Oncology, Queen's University, Kingston, Canada;; 14 Department of Neurosurgery, Sírio Libanês Hospital, São Paulo, Brazil;; 15 Department of Neurosurgery, State University of Campinas, Campinas, São Paulo, Brazil

**Keywords:** Cavernous sinus, Meningiomas, Stereotactic radiosurgery (SRS), Treatment effectiveness

## Abstract

**BACKGROUND AND OBJECTIVES:**

Meningiomas are the most common primary central nervous system tumors, with cavernous sinus meningiomas (CSMs) making up a small fraction (1% of intracranial tumors). CSMs are challenging to treat due to their location and potential invasion. Therapy methods have shifted from aggressive resections to less invasive techniques such as stereotactic radiosurgery, reducing morbidity and improving survival. This study reviews the effectiveness and safety of radiosurgery for CSMs, blending historical and contemporary approaches to guide future treatments.

**METHODS:**

A search was performed in MEDlINE, Embase, and Cochrane databases, following Cochrane and Preferred Reporting Items for Systematic Reviews and Meta-Analysis guidelines. Eligible studies included randomized or observational studies with ≥4 patients reporting on radiosurgery for CSM. The random-effects model was used to calculate a single proportion analysis with 95% CIs. Statistical analyses were performed using RStudio.

**RESULTS:**

Twenty seven studies, encompassing 1851 patients, were included in this analysis, with a population median age of 27 years. Outcomes were assessed as follows: the 10-year progression-free survival rate was 87% (323 patients; 95% CI: 73%-100%; I^2^ = 89.6%), clinical deterioration occurred in 6% of cases (957 patients; 95% CI: 3%-8%; I^2^ = 71.3%), clinical improvement was observed in 35% of patients (1106 patients; 95% CI: 27%-43%; I^2^ = 86.6%), major complications rate was 4% (688 patients; 95% CI: 1%-6%; I^2^ = 71.8%), and minor complications was 2% (653 patients; 95% CI: 0%-3%; I^2^ = 68.6%).

**CONCLUSION:**

This study reveals stereotactic radiosurgery as an effective treatment of CSMs, showing high long-term progression-free survival and significant tumor control with few complications.

**ABBREVIATIONS:**
CSM: cavernous sinus meningiomas
GK: Gamma Knife
LINAC: linear accelerator
RT: radiotherapy
SRS: stereotactic radiosurgery.

Meningiomas are the most common primary tumors of central nervous system, accounting for 40.4% of all tumors of central nervous system.^1,2^ The incidence of skull base meningioma is 2 per 100 000 persons per year.^2^ However, cavernous sinus meningioma (CSM) incidence is notably low, representing only 1% of all intracranial tumors, and is predominantly found in women in their third or fourth decade of life.^2,3^ Owing to the rarity of CSM, there are limited clinical studies on the subject, underscoring the need for comprehensive data on its clinical features, treatment approaches, and long-term prognosis.^4,5^

The cavernous sinus (CS) is an anatomic treasure chest beneath the temporal lobe within the middle cranial fossa, stretching from the superior orbital fissure to Meckel cave.^6^ CSM frequently extends into the spheno-orbital region beyond the CS, potentially causing visual deterioration, eye protrusion, and neurovascular damage due to direct pressure or secondary effects of bony overgrowth.^6^ CSMs can develop within the CS or spread from nearby areas, including the petrous bone, anterior clinoid process, petroclival region, or sphenoid wing.^7^ Although these lesions generally progress slowly and are not life-threatening, CSMs can still be unpredictable, as clinical symptoms do not always correlate with tumor size or growth rate.^8^

The CS was first surgically approached by Parkinson in 1965.^9,10^ Although multiple transcranial routes have been described for accessing the CS, these procedures are associated with high morbidity due to the complex anatomy and the presence of critical neurovascular structures.^9,11-14^ As a result, those increasingly aggressive surgical resections for CSMs eventually gave way to more conservative strategies.^12-16^ Stereotactic radiosurgery (SRS) and fractionated stereotactic radiotherapy have since emerged as less invasive alternatives, offering lower morbidity and comparable, or even superior, overall survival rates.^15,16^

Therefore, SRS is a minimally invasive approach for treating benign tumors infiltrating the CS, offering long-lasting results.^16^ It can improve cranial nerve (CN) deficits and offers an adjuvant option for residual lesions that show progression in this area.^17^ Depending on the location, the dose of SRS (in Gy) may need to be modulated to avoid damage to adjacent structures that are more sensitive, such as the CNs.^18^ Factors such as tumor location, size, extent, grade, symptomatology, and patient age guide treatment decisions.^18^ The literature presents mounting proof that SRS is successful in attaining prolonged tumor management; however, there is still a lack of robust evidence when compared with other treatment modalities.^19-21^ The aim of this study was to assess the efficacy and safety of radiosurgery in CSMs.

## METHODS

This systematic review and meta-analysis was conducted in accordance with the Preferred Reporting Items for Systematic Reviews and Meta-Analysis guidelines. The study protocol was not registered in any registry. All data analyzed were extracted directly from the full text and supplementary materials of previously published studies, publicly available or accessible through subscription.

### Eligibility Criteria

This study review and meta-analysis followed these inclusion criteria: (1) studies published in English, (2) randomized or observational studies, (3) original data sets, (4) ≥4 patients in the interest group, and (5) reporting on SRS for CSMs. Studies were excluded if they were (1) case reports, conference abstracts, reviews, technical notes, editorials, or letters, or (2) duplicated findings from other studies.

### Search Strategy

We conducted a comprehensive search for clinical outcomes following radiosurgery for CSM across MEDlINE, Embase, Cochrane, and Web of Science. Search terms included “cavernous sinus,” “cavernous sinus meningiomas,” “CSM,” “radiosurgery,” “gamma knife,” “stereotactic,” “linear accelerator,” and “CyberKnife,” with abbreviations. Rayyan Software 22 was used for duplicate screening.^22^

### Screening

Search results were imported into Rayyan QCRI software for manual duplicate removal and initial title and abstract screening by 2 independent reviewers. The senior author resolved disagreements. Selected studies then underwent full-text screening by 2 independent reviewers, with conflicts resolved by a third reviewer.

### Outcomes Definitions and Data Extraction

Outcomes were assessed based on the classifications provided by the included studies. Symptoms were evaluated by CN deficits (I-XII), visual impairment (CN II—visual acuity and fields), extraocular neuropathy (CN III, IV, VI—ophthalmoplegia, diplopia), and trigeminal neuropathy (neuralgia, dysesthesia). Clinical improvement was defined as the resolution or partial recovery of at least 1 preexisting CN or neurological deficit. Clinical deterioration was defined as either a subjective worsening of an existing CN or neurological deficit or the development of a new deficit. Tumor progression, stabilization, and regression were defined radiologically as >25% increase, <25% change, and >25% reduction in tumor volume on MRI, respectively. Some studies used different cutoff values for tumor size variation, such as 10%^23-25^ and 50%.^26^ Overall survival was typically defined as the time from the initial SRS treatment to death from any cause, whereas progression-free survival (PFS) was defined as the time from SRS to the first documented tumor progression.

Follow-up complications refer to those occurring between the end of radiosurgery and the last follow-up recorded in the studies. Endocrinological dysfunctions were considered when pertained to clinical symptoms and laboratory values. We included transient diabetes insipidus, hyperprolactinemia, hypopituitarism, and panhypopituitarism. Owing to significant heterogeneity in studies' definition of complications was defined as any postoperative situation necessitating further intervention. Major complications were defined as being potentially life-threatening or if they brought permanent deficits to the patient, whereas minor complications were defined as not potentially life-threatening or not bringing permanent deficits to the patients.

### Risk-of-Bias Assessment

The included studies' bias was assessed using the Risk of Bias in Nonrandomized Studies—of Interventions tool.^27^

### Statistical Analysis

This meta-analysis was performed using the Cochrane Collaboration and the Preferred Reporting Items for Systematic Reviews and Meta-Analysis statement guidelines. Proportions with 95% CIs were used to compare outcome treatment effects. I^2^ statistics were used to assess for heterogeneity; *P* values inferior to .05 and I^2^ < 35% were considered significant. Statistical analysis was performed using RStudio software (version 4.2.3, R Foundation for Statistical Computing). Baujat plots were plotted with all studies pertaining to each analysis, but only the 5 most influential studies received in-plot legends, for the sake of visual clarity.

## RESULTS

### Study Selection and Characteristics

#### Study Selection

**FIGURE 1.**
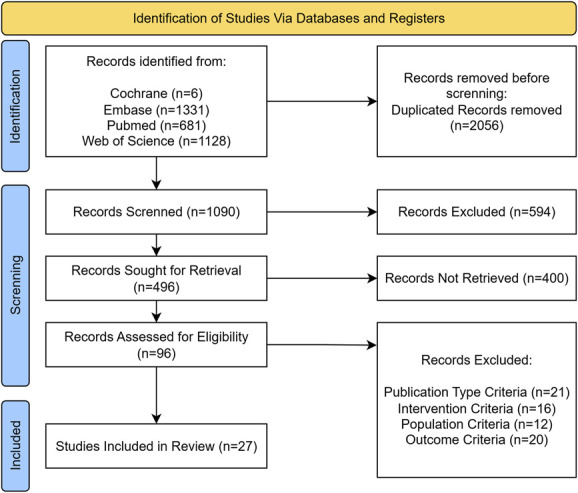
Preferred Reporting Items for Systematic Reviews and Meta-Analysis flow diagram.

On searching MEDLINE, Web of Science, Embase, and Cochrane, 3146 studies were identified. After the removal of duplicates, 1090 studies were screened based on title and abstract. Ninety-six studies were then selected for full-text review based on the inclusion criteria presented. Finally, 27 studies were included in our analysis. The study selection process is further presented in Figure 1.

#### Risk-of-Bias Assessment

**FIGURE 2.**
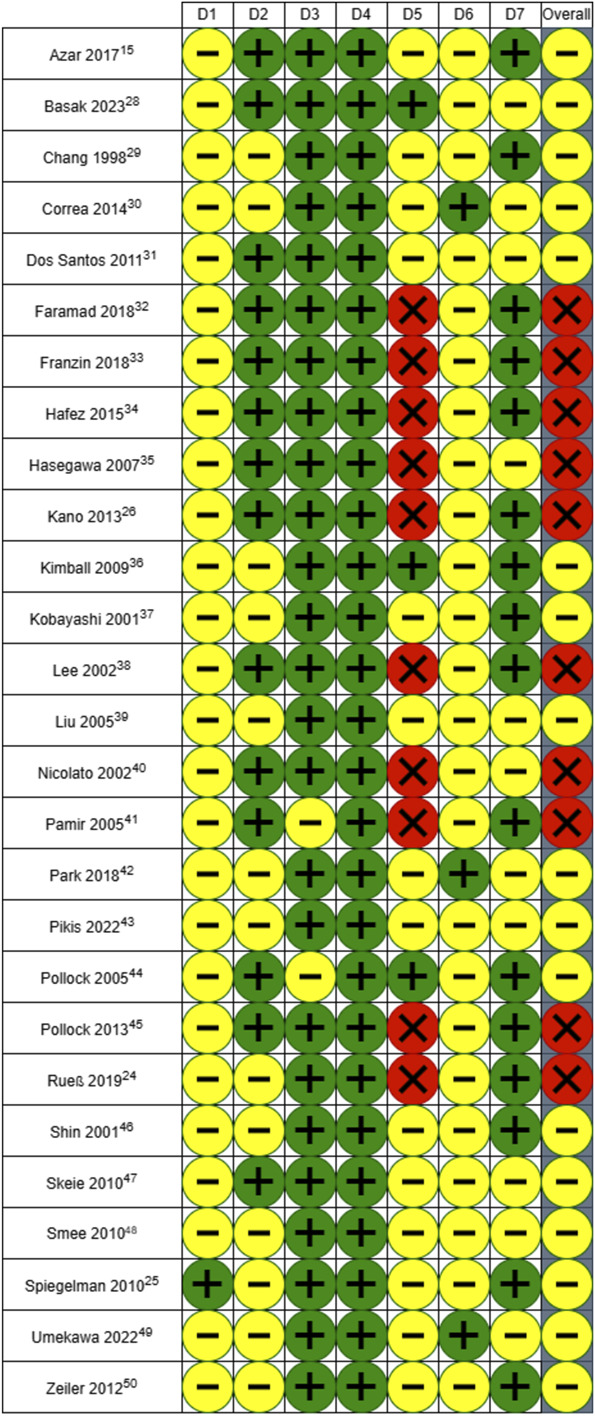
“Traffic light” plot of the domain-level judgments for each individual result.

**FIGURE 3.**
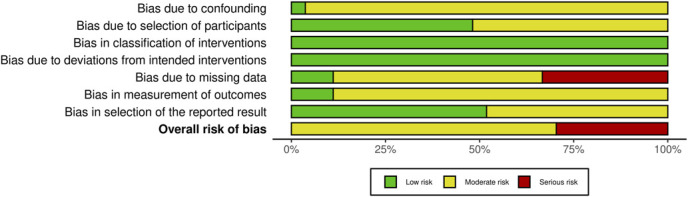
Overall weighted bar plot of risk-of-bias judgments within each bias domain.

The results of the risk-of-bias assessment are presented in Figures 2 and 3.

#### Baseline Characteristics

The data in this meta-analysis derive from 27 studies assessing the use of SRS in treating CSM, including a total of 1851 patients. The median patient age was 55 years, ranging from 7 to 93 years. Male patients constituted approximately 11.6% of the cohort, whereas female patients made up 34.1%, and 54.3% were not reported. The mean tumor volume was 7.34 cm^3^. Previous surgeries were reported in 205 patients (11.1% of the cohort). The most common presenting symptom was CN deficits (79%), followed by extraocular neuropathy (55%), trigeminal neuropathy (31%), and visual acuity impairment (13%). Gamma Knife (GK; The Gamma Knife, Elekta AB) was the most commonly used SRS modality (74.1%), followed by linear accelerator (LINAC) (22.2%) and CyberKnife (3.7%). The mean marginal dose ranged from 6 to 22.5 Gy, with an average of 13.65 Gy. The mean follow-up duration was approximately 53,24 months, ranging from 1 to 300 months. These data provide insights into patient demographics, treatment modalities, and post-treatment monitoring in the analyzed studies. Baseline characteristics of the populations in each study are presented in Table.

**TABLE.** Patients Baseline Characteristics

Study	Total sample	Mean age, y (range)	M/F	Previous surgery, n (%)	CN deficits, n (%)	Imaging-defined CSM, n (%)	Modality	Mean marginal dose, Gy	Mean follow-up, mo	Mean tumor volume, cm^3^	Previous radiotherapy
Azar et al^15^	122	50.9 (16-81)^a^	NR	0	NR	122 (100)	SRS/GK	13 (6-18)^a^	32.4 (6-120)^a^	9.75	NR
Basak et al^28^	11	54.9 (32-77)	3/8	0	8 (72.7)	11 (100)	SRS/GK	11.7 (10-14)	15	6.63 (2.8-14.2)	0
Chang et al^29^	24	47.8 (28-78)	NR	16 (67)	13 (54)	24 (100)	SRS/LINAC	17.7 (14-20)	45.6 (19-80)	6.83 (0.45-22.45)	NR
Correa et al^30^	32	61.03 (39-107)	NR	NR	NR	NR	SRS/LINAC	14 (13-15)^b^	73^a,b^	8.25 (1.5-58.7)	NR
dos Santos et al^31^	47	51.6 (16-90)^a^	NR	0	NR	NR	SRS/LINAC	13.95 (13-16)^a^	86.8 (17.1-179.4)^a^	3.7	NR
Faramand et al^32^	149	57 (21-93)^b^	37/112	0	149 (100)	149 (100)	SRS/GK	13 (9-20)^b^	58 (12-300)	7 (0.5-37.5)^b^	0
Franzin et al^33^	82	62.6 (31-86)^a^	NR	0	NR	81 (100)	SRS/GK	13.8 (10-20)^a^	36^a,b^	7.99 (0.7-30.5)^a^	NR
Hafez et al^34^	51	48 (26-74)^a,b^	NR	0	51 (100)	51 (100)	SRS/GK	NR	36 (24-96)^a^	5.7 (1.8-12.4)^a^	0
Hasegawa et al^35^	49	55 (15-80)^a^	NR	0	NR	49 (100)	SRS/GK	13 (7.5-17)^a^	62 (8-144)^a,b^	13.8 (0.6-121.8)^a^	NR
Kano et al^26^	173	57 (21-86)^b^	NR	0	145 (84)	173 (100)	SRS/GK	13 (10-20)^b^	57.2 (6-185)^b^	7.7 (0.1-37.5)^b^	NR
Kimball et al^36^	55	49 (23-79)^b^	21/34	12 (21.8)	44 (80)	NR	SRS/LINAC	12.86 (10-17.5)^b^	50 (6-169)^b^	5.9 (0.8-17.1)^b^	0
Kobayashi et al^37^	27	52	6/21	17 (63)	NR	NR	SRS/GK	13.6	20.2	13.6	NR
Lee et al^38^	83	59 ± 14^b^	NR	0	83 (100)	83 (100)	SRS/GK	13 ± 2^b^	34.8^a,b^	6.3 ± 5.13^b^	NR
Liu et al^39^	88	NR	NR	NR	NR	NR	SRS/GK	12.1 (8-16)	32.5 (1-84)	6.62 (0.32-17.6)	NR
Nicolato et al^40^	81	56.1 (25-86)^a^	NR	0	NR	NR	SRS/GK	14.6 (11-22.5)^a^	48.9 (12.3-99.1)^a,b^	8.3 (1-20)^a^	0
Pamir et al^41^	26	51.9 (29-74)	7/19	0	NR	NR	SRS/GK	NR	39.6	NR	0
Park et al^42^	120	57 (22-83)^a,b^	NR	NR	NR	NR	SRS/GK	13 (10-20)^a,b^	48.9 (4.8-120)^b^	7.3 (0.1-36.9)^b^	NR
Pikis et al^43^	37	55.05 ± (11.56)	29/8	NR	0	37 (100)	SRS/GK	12.27 ± 2.3	Clinical 66 (IQR 84)^b^	5.73 ± 4.36	NR
									Radiological 72 (IQR 84)^b^		
Pollock et al^44^	49	55.5 (23-82)	11/38	0	38 (77.5)	49 (100)	SRS/GK	15.9 (12-20)	58 (16-144)	10.2 (1.3-35.4)	0
Pollock et al^45^	69	54 (23-82)^b^	15/54	0	61 (88.4)	NR	SRS/GK	16 (12-20)^a^	89 (12-251)^a^	9.3 (1.3-42.2)^a,b^	0
Rueß et al^24^	116	54 (33-82)^b^	25/91	41 (35.3)	75 (64.6)	75 (65)	SRS/LINAC or CK	12.6 (11-18)^b^	Clinical 55 (3-266)^b^	5.7 ± 3.3 (0.6-16.2)	NR
									Radiological 54 (3-266)^b^		
Shin et al^46^	40	50.5 (26-73)^b^	11/29	NR	NR	12 (30)	SRS/GK	18 (12-22.5)^b^	42 (12-123)^b^	4.3 (0.7-25.7)^b^	NR
Skeie et al^47^	40	58.2 ± 14.9	NR	0	33 (82.5)	37 (92.5)	SRS/GK	12.5 ± 1.6	75.9 ± 61.1	6.8 ± 5.6	0
Smee et al^48^	57	54 (7-82)^a^	NR	NR	1 (1.8)	NR	SRS/LINAC	14^b^	37^b^	5.5^b^	NR
Spiegelmann et al^25^	102	57 (31-86)	30/72	35	101 (99)	67 (65.7)	SRS/LINAC	13.5 (12-17.5)	67 (12-180)	7	NR
Umekawa et al^49^	91	54 (47-62)	20/34	72 (79)	69 (76)	NR	SRS/GK	16 (14-18)^b^	89 (44-176)	5.2 (3.3-11.3)	NR
Zeiler et al^50^	30	55.1 (29-79)	8/22	12 (40)	26 (86.6)	NR	SRS/GK	13.5 (12.5-15)	36.1 (3-80)	7.9 (3.25-16.1)	0

CK, CyberKnife; CN, cranial nerve; CSM, cavernous sinus meningioma; F, female; GK, Gamma Knife; M, male; NR, not reported; SRS, stereotactic radiosurgery.

a Data gathered from hole study population, not only from interest group.

b Median.

### Pooled Analysis of Studies

**FIGURE 4.**
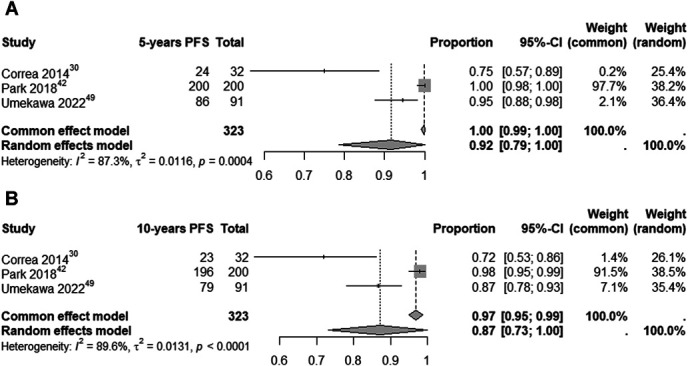
Analysis of PFS at 5 **A** and 10 years **B** of follow-up in patients undergoing SRS for meningioma treatment. PFS, progression-free survival; SRS, stereotactic radiosurgery.

In the pooled analysis of PFS at 5 and 10 years of follow-up, 3 studies with a total of 323 patients reported quantitative data on events. The analysis showed an estimated PFS rate of 92% (95% CI: 79%-100%; I^2^ = 87.3%, random-effects model) at 5 years post-SRS. At 10 years, the estimated PFS rate was 87% (95% CI: 73%-100%; I^2^ = 89.6%, random-effects model), as shown in Figure 4.

**FIGURE 5.**
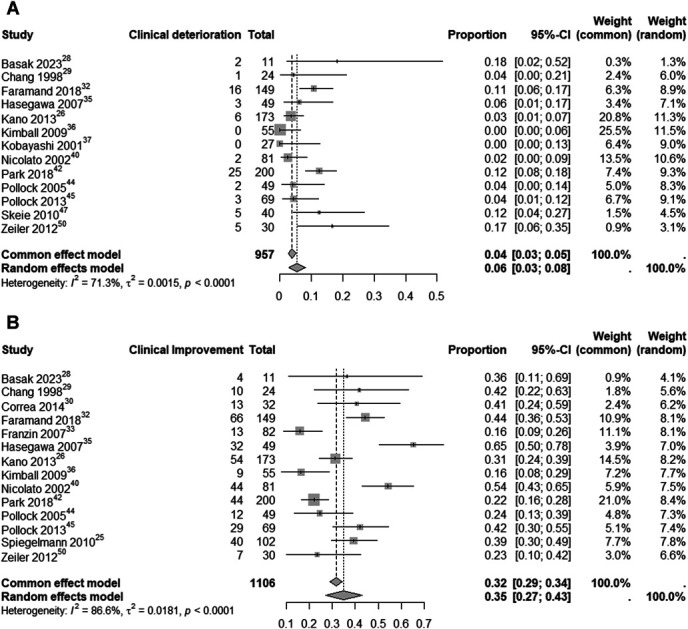
Clinical deterioration **A** and clinical improvement **B** rate analysis in patients undergoing SRS for CSMs. CSM, cavernous sinus meningioma; SRS, stereotactic radiosurgery.

**FIGURE 6.**
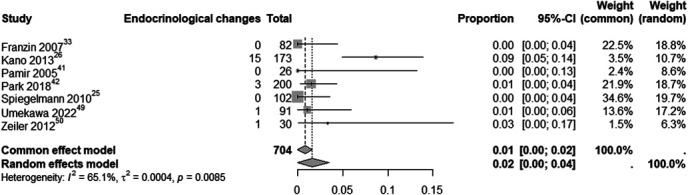
Analysis of endocrinological changes among CSM patients treated with SRS. CSM, cavernous sinus meningioma; SRS, stereotactic radiosurgery.

An analysis of clinical deterioration and improvement rates in patients treated with SRS for CSMs included 957 participants. The estimated clinical deterioration rate after SRS was 6% (95% CI: 3%-8%; I^2^ = 71.3%; random-effects model, Figure 5). By contrast, the estimated clinical improvement rate was 35% (95% CI: 27%-43%; I^2^ = 86.6%; random-effects model, Figure 5). In addition, 7 studies involving 704 patients analyzed endocrinological changes specifically, with an estimated rate of 2% (95% CI: 0%-4%; I^2^ = 65.1%; random-effects model), as shown in Figure 6.

**FIGURE 7.**
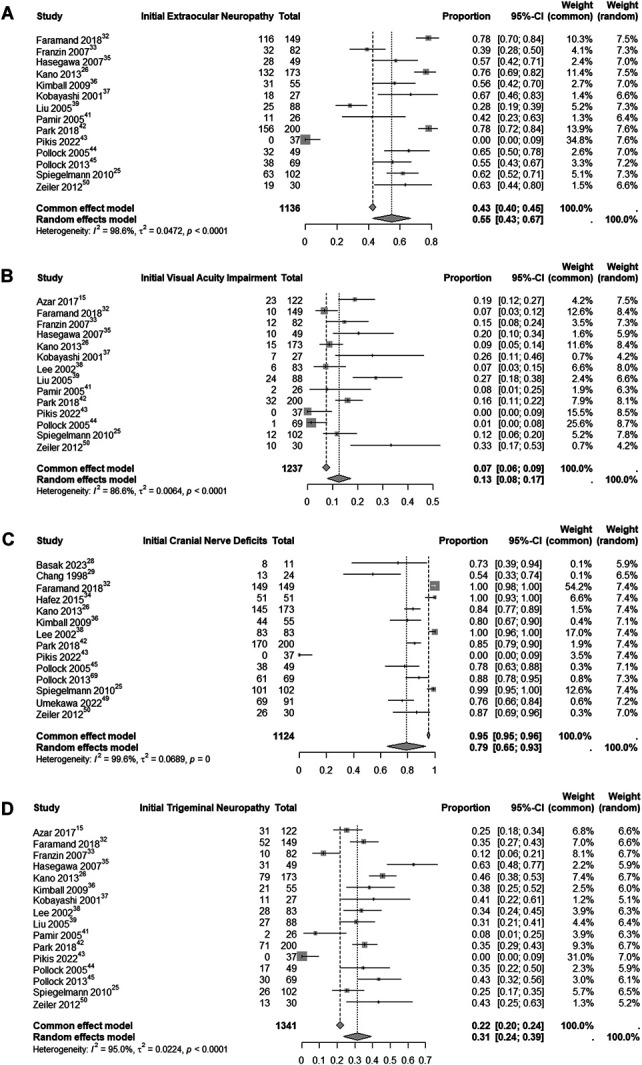
Analysis of initial neurological deficits (extraocular neuropathy **A**, visual acuity impairment **B**, CN deficits **C**, and trigeminal neuropathy **D**) among CSM patients. CN, cranial nerve; CSM, cavernous sinus meningioma.

**FIGURE 8.**
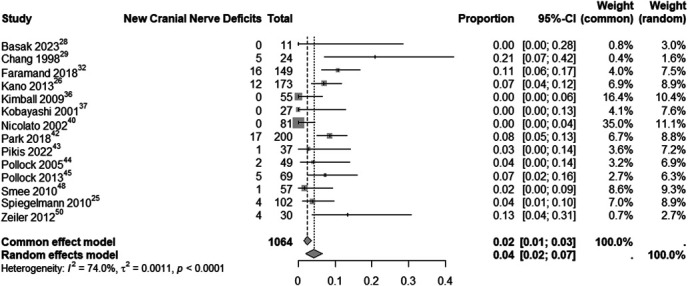
Development of new CN deficits after intervention (SRS) in CSM patients. CN, cranial nerve; CSM, cavernous sinus meningioma; SRS, stereotactic radiosurgery.

In evaluating initial neurological deficits at presentation in patients with CSMs, we conducted 4 analyses of quantitative data for each specific deficit, as shown in Figure 7. We also analyzed the development of new CN deficits postintervention, with an estimated rate of 4% (95% CI: 2%-4%; I^2^ = 74%; random-effects model), as shown in Figure 8.

**FIGURE 9.**
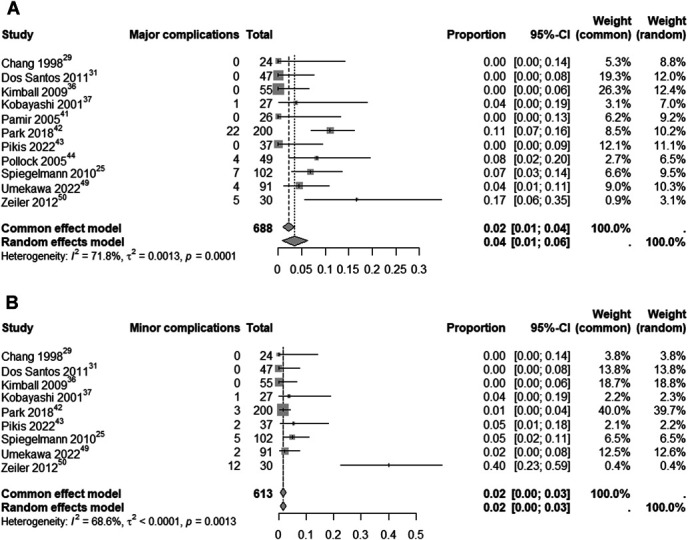
Analysis of the rate of ‘major’ **A** and ‘minor’ **B** complications as a result of SRS in patients with CSMs. CSM, cavernous sinus meningioma; SRS, stereotactic radiosurgery.

For complications, we analyzed major and minor complications related to SRS as the primary treatment of CSM. The analysis of major complications, derived from 11 studies with 688 patients, showed an estimated rate of 4% (95% CI: 1%-6%; I^2^ = 71.8%; random-effects model). The analysis of minor complications, from 9 studies with 613 patients, showed an estimated rate of 2% (95% CI: 0%-3%; I^2^ = 68.6%; random-effects model). Both analyses are depicted in Figure 9.

**FIGURE 10.**
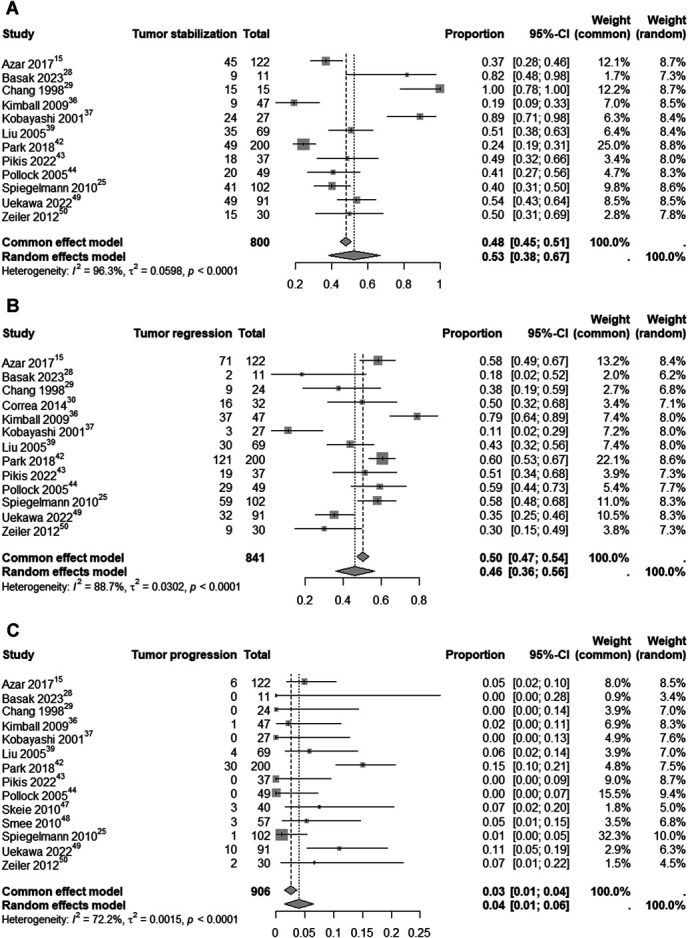
Analysis of tumor stabilization **A**, tumor regression **B**, and tumor progression **C** related to SRS in CSM patients. CSM, cavernous sinus meningioma; SRS, stereotactic radiosurgery.

Analyses were performed for tumor stabilization, regression, and progression in CSM patients undergoing SRS. For tumor stabilization, 12 studies with 800 patients showed an estimated rate of 53% (95% CI: 38%-67%; I^2^ = 96.3%; random-effects model). Tumor regression analysis of 841 patients identified an estimated rate of 46% (95% CI: 36%-56%; I^2^ = 88.7%; random-effects model; Figure 10). Tumor progression data from 906 patients showed an estimated rate of 4% (95% CI: 1%-6%; I^2^ = 72.2%; random-effects model, Figure 10).

**FIGURE 11.**
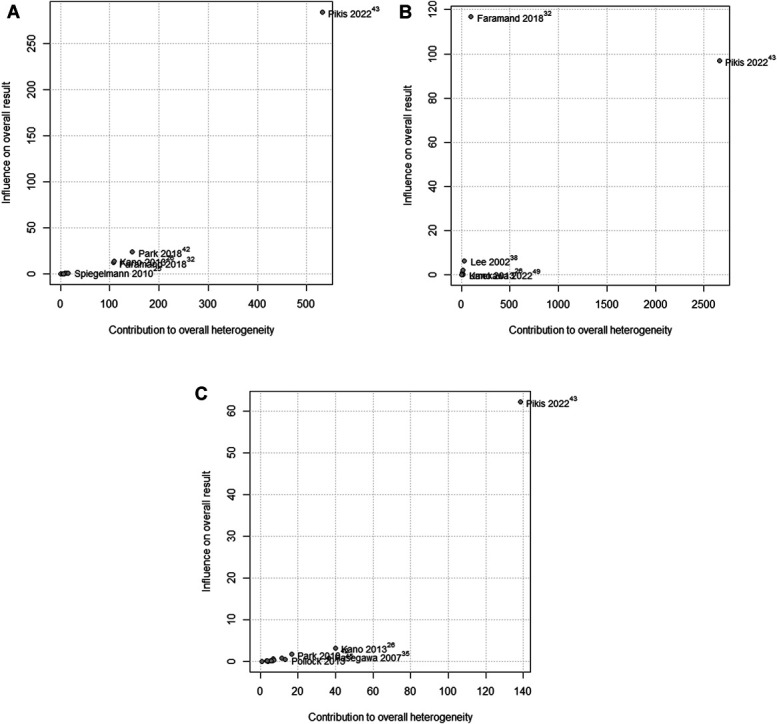
Baujat analysis of initial extraocular neuropathy **A**, initial CN deficits **B**, and initial trigeminal neuropathy **C**. CN, cranial nerve.

A Baujat analysis assessed the influence of included studies on initial extraocular neuropathy, initial CN deficits, and initial trigeminal neuropathy (Figure 11A-11C). The study by Pikis et al^43^ exhibited significant heterogeneity and substantially affected all 3 analyses, affecting both effect size and variability. In the initial CN deficits analysis, Faramand et al^32^ showed high overall contribution but low heterogeneity contribution.

**FIGURE 12.**
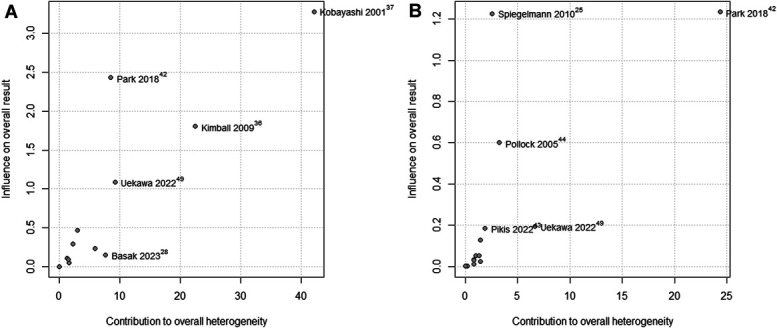
Baujat analysis of tumor regression **A** and tumor stabilization **B**.

A Baujat plot also evaluated the influence of included studies on tumor regression and stabilization (Figure 12A and 12B). For tumor regression, Kobayashi et al^37^ showed high heterogeneity and contribution to overall results, whereas Park et al,^42^ Kimball et al,^36^ and Umekawa et al^49^ had intermediate heterogeneity contributions. For tumor stabilization, Park et al^42^ was most responsible for high heterogeneity, with Umekawa et al,^49^ Pollock et al,^44^ and Spiegelmann et al^25^ showing intermediate heterogeneity contribution.

## DISCUSSION

This systematic review and meta-analysis, encompassing 27 studies with a total of 1763 patients, comprehensively evaluates SRS for CSM. The primary outcomes from the analysis were the following: (1) 5-year and 10-year PFS of 92% and 87%, respectively; (2) tumor control rates of 53% for stabilization, 46% for regression, and 4% for progression; (3) major and minor complication rates of 4% and 2%, respectively; and (4) clinical deterioration and improvement rates after treatment of 6% and 35%, respectively. CSM are a rare subset of intracranial meningiomas that often exhibit benign histology and slow growth, and are seldom life-threatening.^7,51^ However, their location poses significant risks by potentially compressing adjacent nerves and arteries, leading to various symptoms and the possibility of invading crucial neurovascular structures.^6,51^ The challenges and significant morbidity associated with surgical resection have prompted a shift toward more conservative treatment strategies, with radiotherapy (RT) increasingly preferred either as a primary approach or as an adjunct to surgery.^1,52^ This trend reflects a growing preference to minimize morbidity while striving to preserve or improve patient outcomes and quality of life.^52^

This study's pooled analysis of 323 patients highlighted a 5-year PFS of 92% and a 10-year PFS of 87%, underscoring the potential of SRS as an effective treatment for this population. However, notable variance was observed between the outcomes reported by Correa et al^30^ and other studies. This discrepancy may be due to factors such as tumor grade and histology, which significantly affect patient outcomes.^53^

In addition to tumor histology, the variation in outcomes is likely due to different SRS modalities: Correa et al^30^ used LINAC, whereas Park et al^42^ and Umekawa et al^49^ used GK. When analyzing studies using LINAC or GK for various meningioma types and grades, a trend toward superior PFS outcomes is observed in patients treated with GK.^54-57^

Evaluating the feasibility of a therapeutic regimen requires meticulous assessment of clinical outcomes and adverse events. Our findings indicate a low rate of clinical deterioration (6%) and a significant clinical improvement (35%) after SRS. Some authors suggest that patient outcomes are directly linked to tumor growth control and the time from symptom onset to SRS, likely due to the involvement of nearby structures and the relief or worsening of neurovascular and nerve compression.^32,58^ In our study, there is a slight correlation between clinical deterioration (6%) and tumor progression (4%), as well as between clinical improvement (35%) and tumor regression (46%).

Regarding adverse events, we report a 4% incidence of major complications and 2% of minor complications, with a more specific analysis on endocrinological changes, reaching only 2% rate, and CN deficits, reaching only 4% of patients. In SRS, some complications are expected due to radiation affecting nearby radiosensitive structures such as the optic nerve, chiasm, brainstem, and vascular structures.^59-61^ Despite the low incidence of complications, we must consider the potential for progressive and irreversible impacts on patients' quality of life.^59^

As previously noted, surgical treatment of CSM is hazardous and associated with a high rate of morbidity.^8^ Comparing our findings with those from surgical resection, initial results show comparable outcomes between both therapies regarding PFS and recurrence-free survival. However, long-term outcomes appear more favorable with SRS, as seen in the studies by De Jesús et al and Amelot et al.^8,62^ De Jesús et al^62^ showed a PFS of 93.9% and 80.7% at 3 and 5 years after total resection, and a PFS of 87.2% at 3 years and 61.5% at 5 years after subtotal resection. Amelot et al^8^ reported a PFS rate of 90% at 5 years and 82% at 10 years. The differences between these surgical studies are due to advancements in technology and techniques, different surgical approaches, and tumor and patient heterogeneity. Despite these variations, SRS shows approximately a 10% to 20% higher PFS at 10 years compared with surgery. In a recent systematic review from 2018, however, Lee et al^38^ identified a PFS rate at 5 ranging from 86% to 99%, whereas the PFS at 10 years stood in between 69% and 97%.

The complications incidence in SRS has shown to be much lower than in surgery studies, with 4% for major and 2% for minor complications. In the surgical studies examined, complication rates varied between 6% and 33%, whereas mortality rates ranged from 0% to 12%. The variability in mortality and complication rates is particularly pronounced in studies with larger populations and more aggressive surgical approaches, highlighting the risks associated with the intensity of surgical intervention in treating these conditions.^32,56-67^

However, surgery and RT should not be considered mutually exclusive treatments. In some cases, the optimal strategy involves integrating both modalities. Couldwell et al^68^ demonstrated a conservative approach to surgical debulking to reduce tumor volume and decompress the CS before RT. This strategy improved nerve function through decompression and established a safe distance between the optic nerve and the tumor, allowing safer radiosurgery with higher doses and improving overall patient outcomes with low morbidity.

One notable variability between studies is the broad range of Gy doses, typically between 8 and 22 Gy, which correlates with higher tumor volumes, invasiveness, or aggressiveness.

In our meta-analysis, we observed significant heterogeneity among the included studies, which we assessed using a Baujat plot analysis for tumor regression and stabilization. The study by Chang et al^29^ showed the highest heterogeneity in tumor regression, likely due to using imaging instead of biopsy for meningioma confirmation, resulting in variability in patient tumor characteristics.

In examining the heterogeneity observed in tumor stabilization within our meta-analysis, the studies by Kobayashi et al and Kimball et al were notably distinct.^36,37^ The heterogeneity in Kobayashi et al's findings is likely due to the significant inclusion of patients with malignant meningiomas, influencing variability in outcomes. Similarly, the diversity in Kimball et al's study population, which included patients with and without previous surgery, failed surgeries, and recurrent meningiomas, could explain the observed heterogeneity.

### Limitations

Several limitations should be considered when interpreting our findings. The predominance of observational studies introduces potential biases, as these designs may not control for all confounding variables, affecting outcome reliability. Missing data in many studies also pose a high risk of bias, hindering accurate comparisons of baseline characteristics.

The notable heterogeneity in methodologies and SRS types complicates direct comparisons and may skew overall analysis. Some studies did not specify the SRS or RT devices used, which affects result interpretation. Bias may arise from a lack of differentiation between SRS techniques due to their distinct characteristics and efficacy profiles. In addition, variations in demographics, tumor histopathology, underlying health conditions, and previous treatments could act as confounders. Tumor characteristics, including grade, subtype, and molecular features, may influence SRS effectiveness. Last, restricting analysis to meningiomas within or invading the CS may introduce bias, as differences in tumor origin, location, and invasion extent significantly affect behavior and treatment response.

## CONCLUSION

This systematic review and meta-analysis highlight the potential of SRS as an effective treatment of CSM. Our results showed high PFS at 5 and 10 years, significant tumor control, and low complication rate. However, the observed heterogeneity among studies, particularly regarding different SRS techniques, tumor characteristics, and previous treatments, emphasizes the need for standardized methodologies in future research to determine the best approach for each patient.
